# MicroRNAs-mediated regulation of the differentiation of dental pulp-derived mesenchymal stem cells: a systematic review and bioinformatic analysis

**DOI:** 10.1186/s13287-023-03289-5

**Published:** 2023-04-11

**Authors:** Pedram Iranmanesh, Amirhossein Vedaei, Sadra Salehi-Mazandarani, Parvaneh Nikpour, Saber Khazaei, Abbasali Khademi, Kerstin M. Galler, Mohammad-Hossein Nekoofar, Paul M. H. Dummer

**Affiliations:** 1 Dental Research Center, Department of Endodontics, Dental Research Institute, School of Dentistry, Isfahan University of Medical Sciences, Isfahan, Iran; 2 Student Research Committee, School of Dentistry, Isfahan University of Medical Sciences, Isfahan, Iran; 3 Department of Genetics and Molecular Biology, Faculty of Medicine, Isfahan University of Medical Sciences, Isfahan, Iran; 4 Department of Endodontics, School of Dentistry, Kermanshah University of Medical Sciences, Kermanshah, Iran; 5 Department of Conservative Dentistry and Periodontology, University Hospital Erlangen, Erlangen, Germany; 6 Department of Endodontics, School of Dentistry, Tehran University of Medical Sciences, Tehran, Iran; 7 School of Dentistry, College of Biomedical and Life Sciences, Cardiff University, Cardiff, UK; 8 Department of Tissue Engineering, School of Advanced Technologies in Medicine, Tehran University of Medical Sciences, Tehran, Iran; 9 Department of Endodontics, Bahçeşehir University School of Dentistry, Istanbul, Turkey

**Keywords:** Dental pulp, Differentiation, MicroRNA, Noncoding RNA, Regeneration, Regenerative medicine, Stem cell

## Abstract

**Background:**

Human dental pulp-derived mesenchymal stem cells (hDP-MSCs), which include human dental pulp stem cells (hDPSCs) and stem cells from human exfoliated deciduous teeth (SHEDs), are promising cell sources for regenerative therapies. Nevertheless, a lack of knowledge relating to the mechanisms regulating their differentiation has limited their clinical application. microRNAs (miRNAs) are important regulatory molecules in cellular processes including cell differentiation. This systematic review aims to provide a panel of miRNAs that regulate the differentiation of hDP-MSCs including hDPSCs and SHEDs. Additionally, bioinformatic analyses were conducted to discover target genes, signaling pathways and gene ontologies associated with the identified miRNAs.

**Methods:**

A literature search was performed in MEDLINE (via PubMed), Web of Science, Scopus, Embase and Cochrane Library. Experimental studies assessing the promotive/suppressive effect of miRNAs on the differentiation of hDP-MSCs and studies evaluating changes to the expression of miRNAs during the differentiation of hDP-MSCs were included. miRNAs involved in odontogenic/osteogenic differentiation were then included in a bioinformatic analysis. A miRNA-mRNA network was constructed, and Gene Ontology and Kyoto Encyclopedia of Genes and Genomes (KEGG) pathway analyses were performed. A protein–protein interaction (PPI) network was also constructed.

**Results:**

Of 766 initially identified records through database searching, 42 and 36 studies were included in qualitative synthesis and bioinformatic analyses, respectively. Thirteen miRNAs promoted and 17 suppressed odontogenic/osteogenic differentiation of hDP-MSCs. hsa-miR-140-5p, hsa-miR-218 and hsa-miR-143 were more frequently reported suppressing the odontogenic/osteogenic differentiation of hDP-MSCs. hsa-miR-221 and hsa-miR-124 promoted and hsa-miR-140-5p inhibited neuronal differentiation, hsa-miR-26a-5p promoted and hsa-miR-424 suppressed angiogenic differentiation, and hsa-miR-135 and hsa-miR-143 inhibited differentiation within myogenic lineages. A miRNA-mRNA network including 1890 nodes and 2171 edges was constructed. KEGG pathway analysis revealed MAPK, PI3K-Akt and FoxO as key signaling pathways involved in the odontogenic/osteogenic differentiation of hDP-MSCs.

**Conclusions:**

The findings of this systematic review support the potential application of the specific miRNAs to regulate the directed differentiation of hDP-MSCs in the field of regenerative therapies.

**Supplementary Information:**

The online version contains supplementary material available at 10.1186/s13287-023-03289-5.

## Introduction

Mesenchymal stem cells (MSCs) are stromal cells that have two key features, self-renewal and the ability to differentiate along different lineages [1]. MSCs have been isolated from a variety of tissues such as umbilical cord, bone marrow and adipose tissues [2]. Stem cell populations of oral and dental tissues are also considered as MSCs [3]. They are capable of differentiating into several lineages of cells such as osteocytes, chondrocytes, myocytes, adipocytes and neurons [4]. Gronthos and co-workers were the first to report the isolation and characterization of MSCs from the pulp tissue of third molar teeth [5]. Currently, populations of MSCs have also been isolated from other oral tissues such as periodontal ligament, the pulp tissue of human exfoliated primary teeth, dental follicle, gingiva and apical papilla [3].

Human dental pulp-derived MSCs (hDP-MSCs) have been a major focus of attention in the field of regenerative therapies and tissue engineering due to their accessibility, easy isolation through noninvasive procedures, relative genomic stability during in vitro expansion and multi-lineage differentiation potential [4, 6]. hDP-MSCs includes human dental pulp stem cells (hDPSCs) and stem cells from human exfoliated deciduous teeth (SHEDs) which are isolated from the pulp tissue of permanent and deciduous teeth, respectively. They have been applied to a variety of therapies in regenerative dentistry such as regeneration of the dentine–pulp complex, periodontal tissues and alveolar bone [3]. Furthermore, in the field of regenerative medicine, recent studies have revealed their potential as a new treatment choice for systemic diseases such as diabetes, myocardial infarction and neurodegenerative disorders [7]. Nevertheless, incomplete understanding of the mechanisms regulating their differentiation has limited their clinical application [6].

microRNAs (miRNAs) are short noncoding endogenous RNAs [19–25 nucleotides] which serve as important gene expression regulators in a posttranscriptional manner [8]. They suppress translation or induce deadenylation and degradation of target RNAs mostly via binding to their complementary sequences in the 3’ untranslated region (3′-UTR) [9]. They exist abundantly in different cells and are capable of suppressing multiple targets [10]. miRNAs maintain multiple pivotal functions including the regulation of cell proliferation, differentiation and apoptosis [11, 12]. It has been discovered that during the differentiation of MSCs, the expression profile of miRNAs changes [13–15]. Recently, studies have identified multiple miRNAs capable of promoting or suppressing the direct differentiation of hDP-MSCs to a specific lineage of cells [15–17].

As important regulatory molecules, miRNAs have complex interactions with proteins, genes and other noncoding RNAs such as long noncoding RNAs (lncRNAs), through which they regulate cellular and molecular processes [18, 19]. Recently, bioinformatic analyses have been widely used to explore these interactions. Target mRNAs that bind to the miRNAs are identified and a miRNA-mRNA network is constructed. Cellular signaling pathways which are in association with the mRNAs from the miRNAs–mRNAs network are identified by Kyoto Encyclopedia of Genes and Genomes (KEGG) analysis [20]. Furthermore, Gene Ontology (GO) analysis is performed to determine the functional properties of the mRNAs from the miRNAs–mRNAs network. GO includes biological processes (BP), cellular components (CC) and molecular functions (MF). BP refers to the operations or sets of molecular events happing in a cell. CC correlates with cellular and extracellular parts, and MF is related to the elemental activities of the miRNAs or mRNAs at the molecular level [21]. Finally, to determine the interactions between the proteins regulated by the miRNAs, a protein–protein interaction (PPI) network is constructed [22].

Although recent studies have proposed multiple miRNAs promoting/suppressing the differentiation of hDP-MSCs, no study has been conducted to pool these miRNAs utilizing bioinformatic analyses to identify novel signaling pathways and cellular processes which are associated with the differentiation of hDP-MSCs. The present systematic review aims to provide a comprehensive panel of the miRNAs that regulate the differentiation of hDP-MSCs including hDPSCs and SHEDs, and to conduct bioinformatic analyses to pool the data derived from the included studies.

## Methods

### Protocol and registration

A review protocol was developed and registered at Open Science Framework (OSF) Registries (https://doi.org/10.17605/OSF.IO/87VQE). The present systematic review was performed according to the Preferred Reporting Items for Systematic Reviews and Meta-Analyses (PRISMA) Statement [23].

### Review question

What are the miRNAs that regulate (promote or suppress) the differentiation of hDP-MSCs including hDPSCs and SHEDs?

### Eligibility criteria

*Inclusion criteria*:

Experimental studies which assessed the (promotive or suppressive) effect of miRNAs on the differentiation of hDP-MSCs and the studies which assessed changes in miRNAs expression during the differentiation of hDP-MSCs.

*Exclusion criteria*:

1. Studies which applied dental pulp-derived stem cells from non-human species.
2. Studies which did not report differential expression data of mature miRNAs.
3. Studies which were not an original study, with no in vivo or ex vivo results.
4. Literature reviews, book sections, congress summaries, patents, commentaries, methodological approaches, opinion articles, previews and hypothesis articles.
5. Studies which were retracted.
6. Studies which were not in English.
7. Studies which were not available in full text even after attempts to contact authors.

### Literature search strategy

The search strategy was developed (Additional file 1: Table S1) and the literature search was performed in MEDLINE (via PubMed), Web of Science, Scopus, Embase and Cochrane Library up to June 15, 2021, without initial date restriction. ProQuest, OpenGrey, WorldCat and Google Scholar (first 100 hits) were searched for the grey literature. Additionally, hand searching was conducted from reference lists of included studies and relevant published reviews [24–26]. An attempt was made to contact authors via e-mail in case of missing information.

### Screening and data extraction

Initially, duplicate studies were excluded. Two review authors (A.V. and P.I.) screened titles and abstracts independently to identify studies which potentially meet the inclusion criteria. The full texts of retrieved studies were then assessed for eligibility. Disagreements were resolved through discussion with another team member (S.Kh.) to reach a consensus. The included studies were categorized into two groups based on their methodology:

- Group I: Studies which assessed and experimentally validated the (promotive or suppressive) effect of specific miRNA(s) on the differentiation of hDP-MSCs.
- Group II: Studies which investigated expression profile changes of miRNAs during the differentiation of hDP-MSCs by utilizing high-throughput techniques.

Two datasheets were designed to extract data from the included studies in each group. The first sheet consisted of author, year of publication, miRNA(s), type of differentiation, regulatory effect of miRNA(s) on differentiation, cell type, cell source, differentiation induction agent, technique of differentiation assessment, assessed differentiation markers and signaling pathways as well as direct target of miRNA(s). The second sheet consisted of author, year of publication, cell type, cell source, type of differentiation, technique of differentiation assessment, number of up-regulated and down-regulated miRNAs and technique of miRNAs assessment. Two reviewers (P.I. and A.V.) collected the relevant data independently using these datasheets. In case of disagreement, another review author (M.H.N.) was consulted to reach a consensus.

### Bioinformatic analysis

miRNAs that were involved in the differentiation to odontogenic/osteogenic lineages were included in the bioinformatic analysis. miRNAs involved in differentiation to other lineages were not included because the number of them was insufficient for further analysis. All the odontogenic/osteogenic-related miRNAs reported in the studies of group I were included for analysis, as they were all experimentally validated to promote/suppress differentiation. On the contrary, in the studies of group II, by drawing a Venn diagram using FunRich software (version 3.1.3), only those miRNAs reported in at least two studies with the same direction of expression change were selected for analysis.

To identify mRNAs interacting with the miRNAs, the multiMiR R package (version 2.3.0) was utilized in RStudio software (version 1.2.5042) [27]. The miRNAs which were not recognized by the R package were disregarded in the bioinformatic analysis. According to the default of the package, 20% of the highest reliable interactions were retrieved from miRTarBase, miRDB and TargetScan databases [28–30]. Then, weak interactions retrieved from miRTarBase were omitted and only interactions with Target Score > 90 from miRDB and context^++^ score < − 0.6 from TargetScan were selected as miRNA-mRNA interactions for subsequent analysis. A miRNA-mRNA network was constructed using Cytoscape software (version 3.8.1) [31]. Functional analyses including GO and KEGG pathways related to the mRNAs from the miRNA-mRNA network were performed using Enrichr online database [32]. A dot plot representing the results of the KEGG pathway analysis was generated using ggplot2 R package [33]. The STRING online database (version 11.0) was used to establish a protein–protein interaction (PPI) network based on the mRNAs from the miRNA-mRNA network [34]. For this aim, a minimum required interaction score was set as the highest confidence (0.9). The CytoHubba plug-in was used to identify hub genes of the PPI network in the Cytoscape software [35].

## Results

### Study selection

**Fig. 1:**
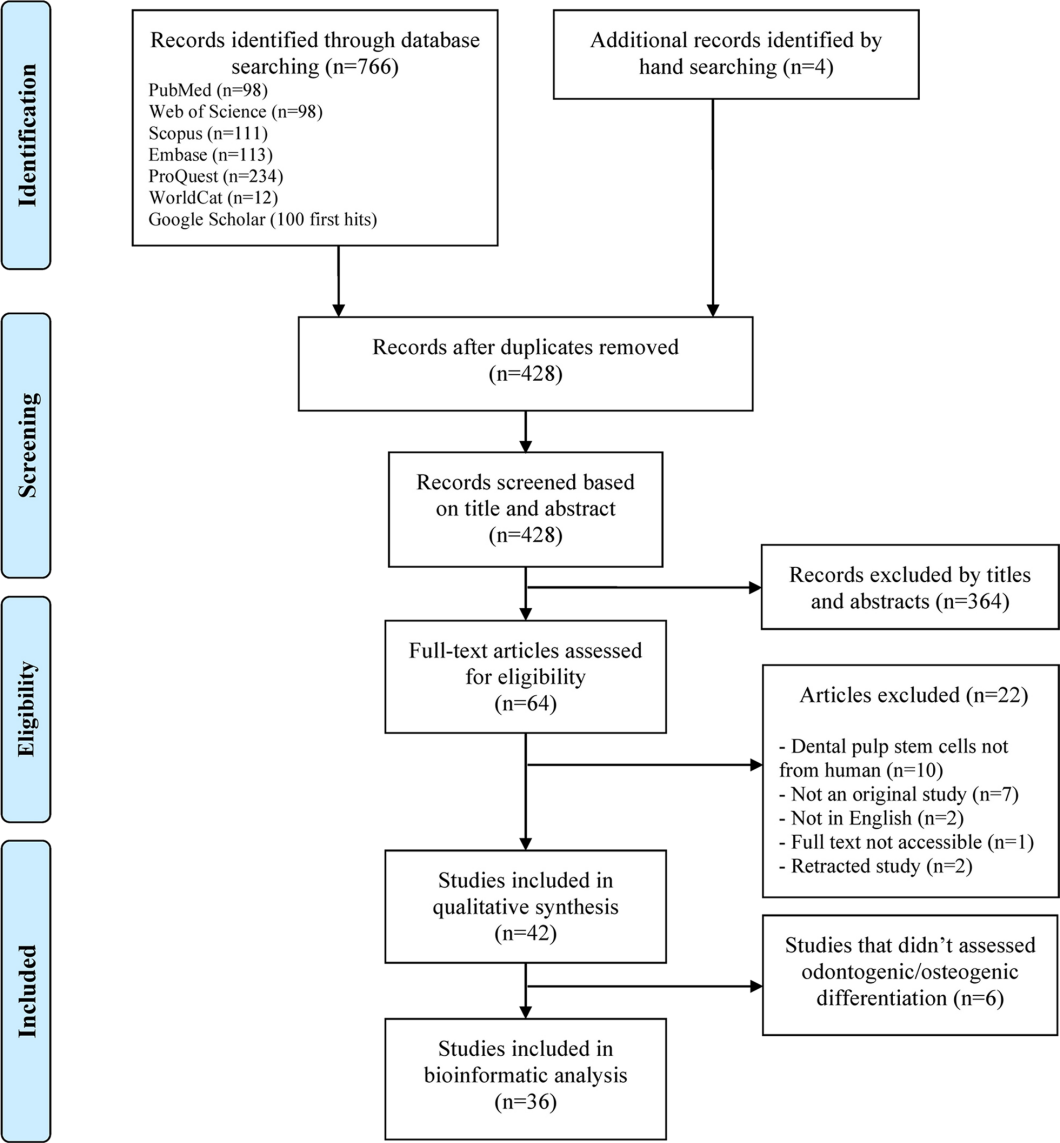
Preferred Reporting Items for Systematic Reviews and Meta-Analyses (PRISMA) flow diagram of the search results and number of records at each stage

The study selection process including the number of studies at each stage is depicted in the PRISMA flow diagram [23] (Fig. 1). In total, 766 studies were identified initially through the electronic search, and 4 additional studies were identified during the manual search. Following further analysis, 342 duplicates were removed, and 364 studies were excluded based on the titles and screening of the abstracts. Finally, after full-text review, a further 22 studies were excluded leaving 42 studies that met the inclusion criteria. A list of excluded studies and reasons for exclusion are reported in Additional file 2: Table S2. Included studies were divided into the following two groups based on their methodology.

### Description of included studies

#### Group I studies

Studies in this group investigated the promotive or suppressive effect of miRNAs on the differentiation of hDP-MSCs by culturing them with growth factors (differentiation inducing agents) and overexpressing or knocking down the miRNAs. In total, 40 studies had assessed the promotive/suppressive effect of specific miRNA(s) on the differentiation of hDPSCs or SHEDs (Table 1), out of which 8 studies [36–43] confirmed their results in vivo in an animal model.

**Table 1:** Main findings of the studies investigating the promotive or suppressive function of miRNAs on the differentiation of hDP-MSCs through overexpressing or knocking down the miRNAs (studies of group I)

Author [reference]	miRNA(s)	Type of differentiation; regulatory effect of miRNA(s)		Cell type	Cell source	Growth factor (differentiation inducing agent)	Technique of differentiation assessment	Assessed differentiation markers	Direct target(s) of miRNA(s)	Signaling pathway
Zhong et al. [36]	hsa-miR-140-5p	Odontogenic/osteogenic	↓	hDPSC	Third molar	AA, DEX, BGP	ARS, ALP activity	DSPP, DMP1, RUNX2, ALP	FGF9, BMP2, lncRNA H19	N/A
Bao et al. [50]	hsa-miR-140-5p	Odontogenic/osteogenic	↓	hDPSC	Premolar	AA, DEX, BGP	ARS, ALP staining, VKS	ALP, DSPP, DMP1, desmoplakin, DLX3	GIT2, lncRNA MALAT1	N/A
Wu et al. [60]	hsa-miR-24-3p	Odontogenic/osteogenic	↓	hDPSC	Third molar	BMP2	ARS	RUNX2, OSX, ALP	TGFBR1, LncRNA LEF1-AS1	N/A
Tu et al. [61]	hsa-miR-30b-3p	Odontogenic/osteogenic	↓	hDPSC	Third molar	AA, DEX, BGP	ARS, ALP staining	RUNX2, DSPP, DMP1, ALP	RUNX2, LncRNA CALB2	N/A
Ji et al. [37]	hsa-miR-188-3p	Odontogenic/osteogenic	↓	hDPSC	Third molar	N/R	ARS, ALP staining	OCN, ALP, OSX	Beclin1, Runx1, CircRNA0026827	Beclin1, Runx1
Ji et al. [38]	hsa-miR-496	Odontogenic/osteogenic	↓	hDPSC	Third molar	AA, DEX, BGP	ARS, ALP staining	RUNX2, OCN, ALP	β-catenin, CircRNA124534	β-catenin
Ge et al. [39]	hsa-miR-617	Odontogenic/osteogenic	↓	hDPSC	Third molar	AA, DEX, BGP	ARS, ALP staining	OSX, RUNX2, ALP	SMAD3, CircRNA SIPA1L1	TGF- β/SMAD
Chen et al.* [15]	hsa-miR-588	Odontogenic/osteogenic	↓	hDPSC	Premolar	AA, DEX, BGP	ARS, ALP staining	N/A	FBN1, LncRNA G043225	N/A
Yang et al. [56]	hsa-miR-143-3p	Odontogenic/osteogenic	↓	hDPSC	Third molar and premolar	AA, DEX, BGP	ARS	ALP, DSPP, BSP, OCN, OPN	RANK	OPG/RANKL
Zhang et al. [40]	hsa-miR-206	Odontogenic/osteogenic	↓	hDPSC	Third molar and premolar	AA, DEX, BGP	ARS, ALP staining	RUNX2, OCN, ALP	CircAKT3	N/A
Xie et al. [97]	hsa-miR-31	Odontogenic/osteogenic	↓	hDPSC	Third molar	AA, DEX, BGP	ARS, ALP staining	RUNX2, OCN, COL1	CircLPAR1	N/A
Liao et al. [41]	hsa-miR-3658	Odontogenic/osteogenic	↓	hDPSC	N/R	BMP2	ARS, ALP activity	RUNX2, OSX, ALP	Long intergenic noncoding RNA 00968	N/A
Lu et al. [51]	hsa-miR-140-5p	Odontogenic/osteogenic	↓	hDPSC	Third molar	AA, DEX, BGP	ARS	DSPP, DMP1	Wnt1	Wnt/β-catenin
Zhong et al. [53]	hsa-miR-218	Odontogenic/osteogenic	↓	hDPSC	Third molar and premolar	N/A^a^	ELIZA (COL1, OCN, OPN)	COL1, OPN, OCN	LncRNA CCAT1	N/A
Chang et al. [54]	hsa-miR-218	Odontogenic/osteogenic	↓	hDPSC	Third molar	AA, DEX, BGP	ARS	N/A	N/A	MAPK/ERK
Wang et al. [57]	hsa-miR-143-5p	Odontogenic/osteogenic	↓	hDPSC	N/R	AA, DEX, BGP	ARS, ALP staining	DSPP, ALP, OCN	MAPK14	P38 MAPK
Liu et al. [98]	hsa-miR-508-5p	Odontogenic/osteogenic	↓	hDPSC	Third molar	AA, DEX, BGP	ARS, ALP activity	DMP1, DSPP, OCN, ALP	GPNMB	N/A
Zhang et al. [58]	hsa-miR-143	Odontogenic/osteogenic	↓	hDPSC	Third molar	AA, DEX, BGP	ALP activity, ALP staining	ALP, BMP2, RUNX2, COL1	TNF-α	NF-κB
Liu et al. [87]	hsa-let-7c	Odontogenic/osteogenic	↓	hDPSC	Third molar	AA, DEX, BGP	ARS	OCN, OSX, DSPP, RUNX2, ALP, COL1, DMP	IGF-1R	JNK/P38 MAPK
Sun et al. [52]	hsa-miR-140-5p	Odontogenic/osteogenic	↓	hDPSC	Third molar	AA, LPS	ARS, ALP staining	N/A	TLR-4	LPS/TLR-4
Song et al.* [17]	hsa-miR-135b	Odontogenic/osteogenic	↓	hDPSC	Third molar	AA, DEX, BGP	ARS	DSPP, DMP1	Smad4, Smad5	BMP2(Smad5/Smad4)
Gay et al.* [55]	hsa-miR-218	Odontogenic/osteogenic	↓	hDPSC	Third molar	AA, DEX, BGP	ARS	RUNX2	RUNX2	N/A
Chen et al. [63]	hsa-miR-216a	Odontogenic/osteogenic	↑	hDPSC	Third molar	AA, DEX, BGP	ARS	DSPP, DMP1	c-Cbl, LncRNA DANCR	N/A
Zhao et al. [62]	hsa-miR-543	Odontogenic/osteogenic	↑	hDPSC	Third molar	AA, DEX, BGP	ARS, ALP activity	OSX, OPN, OCN, RUNX2, ALP	SMURF1, LncRNA MEG3	SMURF1/ RUNX2
Wang et al. [99]	hsa-miR-125a-3p	Odontogenic/osteogenic	↑	hDPSC	Third molar	AA, DEX, BGP	ARS, ALP staining	ALP, DSPP, DMP1	Fyn	NF-κB, β-catenin
Qiu et al. [75]	hsa-miR-146a-5p	Odontogenic/osteogenic	↑	hDPSC	Third molar and premolar	AA, DEX, BGP	ARS, ALP staining	ALP, RUNX2, OSX, DSPP	NOTCH1	Notch
Huang et al. [100]	hsa-miR-223-3p	Odontogenic/osteogenic	↑	hDPSC	Third molar	AA, DEX, BGP	ARS, ALP activity	DSPP, DMP1, ALP	SMAD3	TGF- β1
Hu et al.* [16]	hsa-miR-27a-5p	Odontogenic/osteogenic	↑	hDPSC	Third molar	AA, DEX, BGP	ARS	DSP, DMP1, ALP, RUNX2	LTBP1	TGF-β/SMAD
Zeng et al. [101]	hsa-miR-675	Odontogenic/osteogenic	↑	hDPSC	premolar	AA, DEX, BGP	ARS, ALP staining, ALP activity	DSPP, DMP1, ALP	DLX3^b^	N/A
Xu et al. [59]	hsa-miR-21	Odontogenic/osteogenic	↑	hDPSC	Third molar	TNF-α	ARS, ALP staining	DMP1, DSPP	N/A	STAT3
Ishiy et al. [48]	hsa-miR-1287	Odontogenic/osteogenic	↑	SHED	Human deciduous tooth	N/R	ARS, ALP activity	ALP, RUNX2	N/A	N/A
Dernowsek et al.* [49]	hsa-miR-450a-5p, hsa-miR-28-5p	Odontogenic/osteogenic	↑	SHED	Human deciduous tooth	AA, DEX, BGP	ARS, ALP activity	RUNX2, ALP, BGLAP	STAT1	N/A
Zhang et al. [102]	hsa-miR-633	Odontogenic/osteogenic	↑	hDPSC	Third molar and premolar	N/A^a^	Western blot (COL1, OCN, OPN)	COL1, OPN, OCN	MEPE	N/A
Hara et al.* [64]	hsa-miR-720	Odontogenic/osteogenic	↑	hDPSC	Third molar and premolar	AA, DEX, BGP	ARS, ALP staining	DSPP, OPN, ALP	NANOG	N/A
Mehri-Ghahfarrokhi et al. [45]	hsa-miR-124	Neuronal	↑	hDPSC	N/R	BDNF, N2, B27	Microscopy (morphological changes in cells)	Nestin, SOX2, β-tubulin III, MAP2, peripherin	N/A	
Wen et al. [44]	hsa-miR-221	Neuronal	↑	SHED	Human deciduous tooth	bFGF, EGF, retinoic acid	Immunohistochemistry (MAP2, Nestin, NSE, neurofilament, tyrosine hydroxylase)	MAP2, Nestin, NSE, neurofilament, tyrosine hydroxylase	CHD8	Wnt/β-catenin
Liu et al. [43]	hsa-miR-140-5p	Neuronal	↓	SHED	Human deciduous tooth	B27, FGF, EGF	Western blot (Nestin, βIII‐tubulin)	MAP2, Nestin, βIII‐tubulin, NSE	BMP2, LncRNA C21orf121	N/A
Wu et al. [42]	hsa-miR-26a-5p	Angiogenic	↑	SHED	Human deciduous tooth	N/A^c^	Matrigel assay	VEGF, angiogenin, PDGF, angiopoietin 2	N/A	TGF-β/SMAD2/3
Liu et al. [46]	hsa-miR-424	Angiogenic	↓	hDPSC	Third molar	VEGF	Matrigel assay	vWF, CD31	VEGFR, VEGF	
Li et al. [47]	hsa-miR-143, hsa-miR-135	Myogenic	↓	hDPSC	Third molar	Deoxycytidine	Immunofluorescence microscopy (formation of myotubes)	MOG, Myhc, MEF2C, MYOD	MEF2C, MYOD	N/A

↓Suppressive effect

↑Promotive effect

Studies marked with * are in common with Table 2. They have investigated miRNAs expression profile change in addition with studying the regulatory effect of specific miRNAs on the differentiation of hDP-MSCs

^a^Differentiated cell population was compared with stem cell population

^b^Epigenetically up-regulated by miRNA

^c^SHEDs aggregate-derived exosomes were utilized to induce angiogenic differentiation

AA, Ascorbic acid; ALP, Alkaline phosphatase; ARS, Alizarin red staining; BDNF, Brain-derived neurotrophic factor; bFGF, Basic fibroblast growth factor; BGP, β-glycerolphosphate; BMP2, Bone morphogenetic protein 2; BSP, bone sialoprotein; CHD8, chromodomain helicase DNA-binding protein 8; CircRNA, Circular RNA; COL1, collagen type 1; DEX, Dexamethasone; DLX3, distal-less homeobox 3; DMP1, dentin matrix protein 1; DSP, dentin sialoprotein; DSPP, dentin sialophosphoprotein; EGF, Epidermal growth factor; ERK, Extracellular Receptor Kinase; FBN1, Fibrillin 1; FGF9, Fibroblast growth factor 9; GIT2, G protein–coupled receptor (GPCR)–kinase 2 interacting protein 2; GPNMB, glycoprotein non-metastatic melanomal protein B; hDPSC, Human dental pulp stem cells; IGF-1R, insulin-like growth factor type 1 receptor; lncRNA, Long noncoding RNA; LPS, lipopolysaccharide; LTBP1, Latent Transforming Growth Factor Beta Binding Protein 1; MAP2, Microtubule-associated protein 2; MAPK, mitogen-activated protein kinase; MEF2C, Myocyte Enhancer Factor 2C; MEPE, Matrix extracellular phosphoglycoprotein; Myhc, Myosin heavy chain; MYOD, myoblast determination protein 1; MYOG, Myogenin; N/A, not applicable; NF-κB, nuclear factor kappa light chain enhancer of activated B cells; NOTCH1, Notch homolog 1; N/R, not reported; NSE, neuron-specific enolase; OCN, osteocalcin; OPG: osteoprotegerin; OPN, osteopontin; OSX, osterix; PDGF, Platelet-derived growth factor; RANK, Receptor activator of NF-kB; RANKL, RANK ligand; RUNX, runt-related transcription factor; SHED, Stem cells from human exfoliated deciduous teeth; SMURF1, Smad ubiquitin regulatory factor 1; SOX2, SRY-Box Transcription Factor 2; STAT, signal transducer and activator of transcription; TGF, transforming growth factor; TGFBR1, transforming growth factor-beta (TGF-β) receptor type 1; TLR-4, Toll-like receptor 4; TNF-α, tumor necrosis factor alpha; VEGF, vascular endothelial growth factor; VEGFR, vascular endothelial growth factor receptor; VKS, von Kossa staining; vWF, von Willebrand factor

Differentiation to odontogenic/osteogenic lineages was the most frequently investigated [34 studies], three studies [43–45] investigated neuronal differentiation, two [42, 46] investigated angiogenic (endothelial) differentiation, and one [47] investigated differentiation to myogenic lineage. Regarding the stem cell type, 35 studies had recruited hDPSCs and five studies [42–44, 48, 49] had used SHEDs (Table 1). In all studies of this group, hDPSCs were isolated from human third molar or premolar teeth, and SHEDs were harvested from human deciduous teeth of healthy donors.

Regarding the regulatory function of miRNAs in this group, overall, 16 miRNAs had promotive and 21 miRNAs had suppressive effect on the differentiation: 13 miRNAs promoted and 17 suppressed odontogenic/osteogenic differentiation (Table 1). Among them, hsa-miR-140-5p, hsa-miR-218 and hsa-miR-143 family were more frequently reported suppressing the odontogenic/osteogenic differentiation of hDP-MSCs [36, 50–58]. Two miRNAs (hsa-miR-221 and hsa-miR-124) promoted [44, 45], and one (hsa-miR-140-5p) inhibited neuronal differentiation [43], one (hsa-miR-26a-5p) promoted [42] and another one (hsa-miR-424) suppressed angiogenic differentiation [46], and two (hsa-miR-135 and hsa-miR-143) inhibited differentiation to myogenic lineage [47].

All studies used quantitative reverse transcription polymerase chain reaction (qRT-PCR) to assess miRNAs and dual luciferase reporter assays to verify the direct interaction between a miRNA and its target gene. Techniques for assessing the degree of odontogenic/osteogenic differentiation included alizarin red staining, alkaline phosphatase (ALP) activity assay, ALP staining and von Kossa staining. Furthermore, most of the included studies assessed the expression of differentiation markers to measure the degree of differentiation (Table 1). Markers assessed for odontogenic/osteogenic differentiation were mostly dentin sialophosphoprotein (DSPP), dentine matrix protein 1 (DMP1), runt-related transcription factor 2 (RUNX2), ALP, osteocalcin (OCN), osterix (OSX), osteopontin (OPN) and collagen type 1 (COL1). Regarding neuronal differentiation, assessed markers were nestin, microtubule-associated protein 2 (MAP2), βIII‐tubulin and neuron-specific enolase (NSE) [43–45]. Angiogenic differentiation markers included vascular endothelial growth factor (VEGF), angiogenin, platelet-derived growth factor (PDGF) and von Willebrand factor (vWF) [42, 46]. Myogenic differentiation markers were myocyte enhancer factor 2C (MEF2C), myosin heavy chain (Myhc), myoblast determination protein 1 (MYOD) and myogenin (MYOG) [47].

The direct target genes of miRNAs were determined in 35 studies (Table 1). The direct target gene in all studies except one [59] were suppressed by the miRNAs. In all, 19 studies indicated the signaling pathways through which the miRNAs acted (Table 1). JNK/P38 MAPK, NF-κB, Notch, LPS/TLR-4, OPG/RANKL, SMURF1/RUNX2, TGF-β/SMAD and Wnt/β-catenin were among the reported signaling pathways. In addition to mRNAs, other types of miRNAs targets such as circular RNAs (circRNAs) and lncRNAs were reported. circRNAs included *circRNA0026827*, *circRNA124534*, *circRNA SIPA1L1*, *circAKT3* and *CircLPAR1* [37–40, 97]. Besides, lncRNAs including *H19, MALAT1, LEF1-AS1, CALB2, G043225, CCAT1, LINC00968, MEG3, DANCR* and *C21orf121* were reported as direct targets of miRNAs in 10 studies [15, 36, 41, 43, 50, 53, 60–63].

#### Group II studies

Studies in this group [13–17, 49, 55, 64] [8 studies] used high-throughput techniques including next generation sequencing and microarray to assess and compare the expression profiles of the miRNAs before and after the differentiation (Table 2). Six studies [14, 15, 17, 49, 55, 64] utilized microarrays, and two [13, 16] used sequencing to assess expression profile of miRNAs. qRT-PCR was utilized to validate the results. Regarding the stem cell type, in this group, seven studies [13–17, 55, 64] used hDPSCs, and one [49] recruited SHEDs. Similar to group I studies, all studies of this group isolated hDPSCs from human third molar or premolar teeth, and SHEDs were harvested from human deciduous teeth of healthy donors.

**Table 2:** Main findings of the studies comparing miRNAs expression profile before and after the differentiation (studies of group II)

Author [reference]	Cell type	Cell source	Type of differentiation	Technique of differentiation assessment	Differentially expressed miRNAs	Technique of miRNA assessment
Liu et al. [13]	hDPSC	Third molar	Odontogenic/osteogenic	ARS, ALP staining	113 (63 ↑, 50 ↓)	Sequencing, qRT-PCR
Chen et al.* [15]	hDPSC	Premolar	Odontogenic/osteogenic	ARS, ALP staining	114 (24 ↑, 90 ↓)	Microarray, qRT-PCR
Hu et al.* [16]	hDPSC	Third molar	Odontogenic/osteogenic	ARS	28 (7 ↑, 21 ↓)	Sequencing, qRT-PCR
Song et al.* [17]	hDPSC	Third molar	Odontogenic/osteogenic	ARS	36 (22 ↑, 14 ↓)	Microarray, qRT-PCR
Dernowsek et al.* [49]	SHED	Human deciduous tooth	Odontogenic/osteogenic	ARS, ALP activity	21 (11 ↑, 10 ↓)	Microarray, qRT-PCR
Gay et al.* [55]	hDPSC	Third molar	Odontogenic/osteogenic	ARS	6 (2 ↑, 4 ↓)	Microarray, qRT-PCR
Hara et al.* [64]	hDPSC	Third molar and premolar	Odontogenic/osteogenic	ARS, ALP staining	75 (60 ↑, 15 ↓)	Locked nucleic acid-based Microarray, qRT-PCR
Gong et al. [14]	hDPSC	Third molar and premolar	Odontogenic/osteogenic	ARS, VKS	22 (12 ↑, 10 ↓)	Microarray, qRT-PCR

↓Down-regulated during differentiation

↑Up-regulated during differentiation

Studies marked with * are in common with Table 1. They selected specific miRNA(s) among differentially expressed miRNAs and, as a complementary step, investigated their regulatory effect on the differentiation of hDP-MSCs

ALP, Alkaline phosphatase; ARS, Alizarin red staining; qRT-PCR, Quantitative reverse transcription polymerase chain reaction; VKS, von Kossa staining

Six studies in this group [15–17, 49, 55, 64] selected specific miRNA(s) among differentially expressed miRNAs and, as a complementary step, investigated its regulatory function on the differentiation in the same way as those in group I. These studies were categorized in group I as well. Their results regarding regulatory effects of miRNAs on the differentiation and expression change of miRNAs are depicted in Tables 1 and 2, respectively.

All studies in this group assessed differentiation to odontogenic/osteogenic lineage. Overall, during odontogenic/osteogenic differentiation, 415 miRNAs were reported to be differentially expressed, 181 were up-regulated and 234 were down-regulated (Table 2). The entire list of differentially expressed miRNAs within each study is reported in Additional file 3: Table S3.

### Bioinformatic analysis

**Fig. 2:**
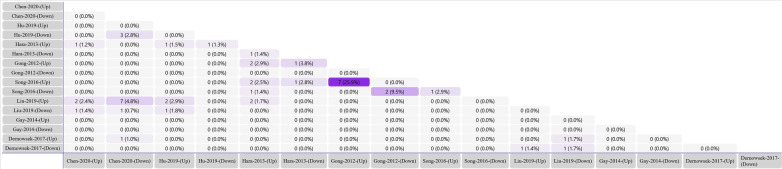
Venn diagram analysis. Cross-tables show the number and percentage of differentially expressed miRNAs which are in common in two corresponding studies. Darker colors represent greater number and percentage of miRNAs

**Fig. 3:**
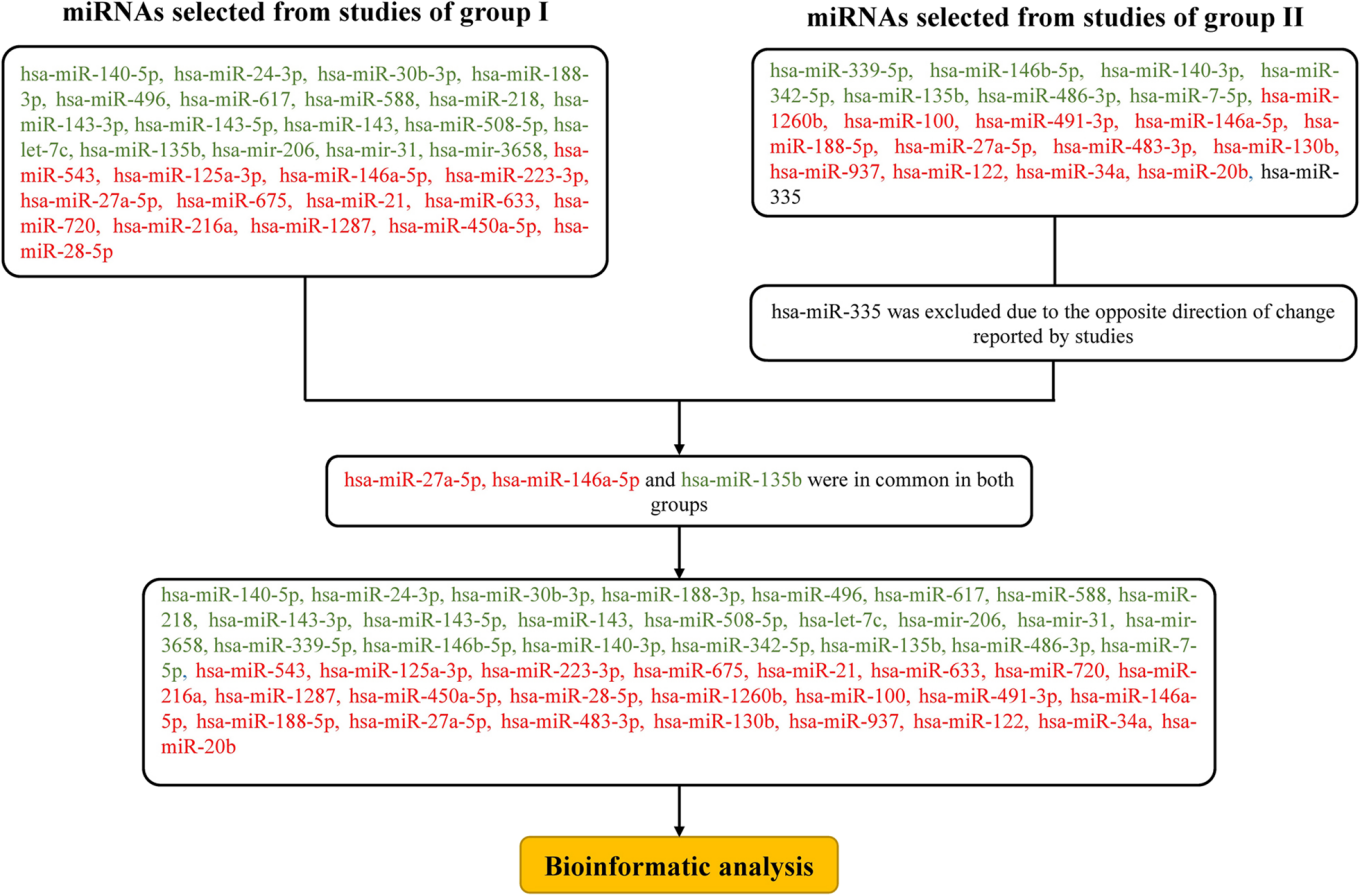
Flowchart of miRNAs selected for bioinformatic analysis. Down-regulated and up-regulated miRNAs are represented by green and red colors, respectively

All the miRNAs in studies of group I which were involved in odontogenic/osteogenic differentiation were included in the analysis. To investigate the miRNAs from the studies of group II, a Venn diagram (Fig. 2) revealed 20 miRNAs reported in at least two studies. Among them, one miRNA (hsa-miR-335) was reported in three studies [14, 17, 64], but with the opposite direction of expression change, which was excluded from the analysis. Overall, 49 miRNAs were retrieved (30 miRNAs from studies of group I and 19 miRNAs from studies of group II); among them, three miRNAs (hsa-miR-27a-5p, hsa-miR-146a-5p and hsa-miR-135b) were duplicated in both groups. Finally, 46 miRNAs were included for bioinformatic analysis (Fig. 3).

**Fig. 4:**
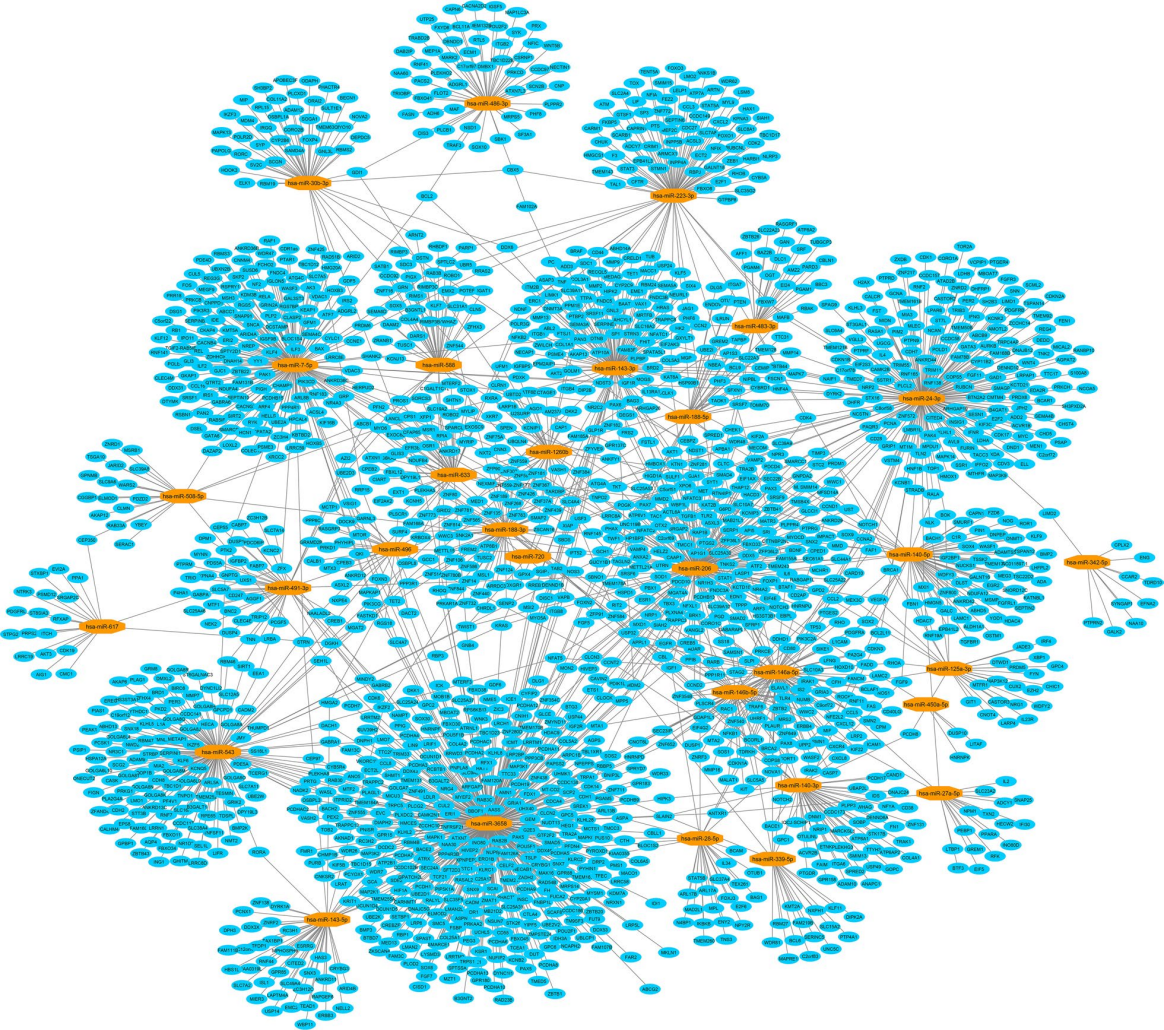
miRNA-mRNA network. Blue ellipses and orange octagons represent mRNAs and miRNAs, respectively. High-resolution network is available on https://www.ndexbio.org/#/network/3a7985b0-cfb7-11ec-b397-0ac135e8bacf?accesskey=f3527730ff25ef6ad91254fb3443e1e96b0a9e49abee3324410b5f17c1b46af6

**Fig. 5:**
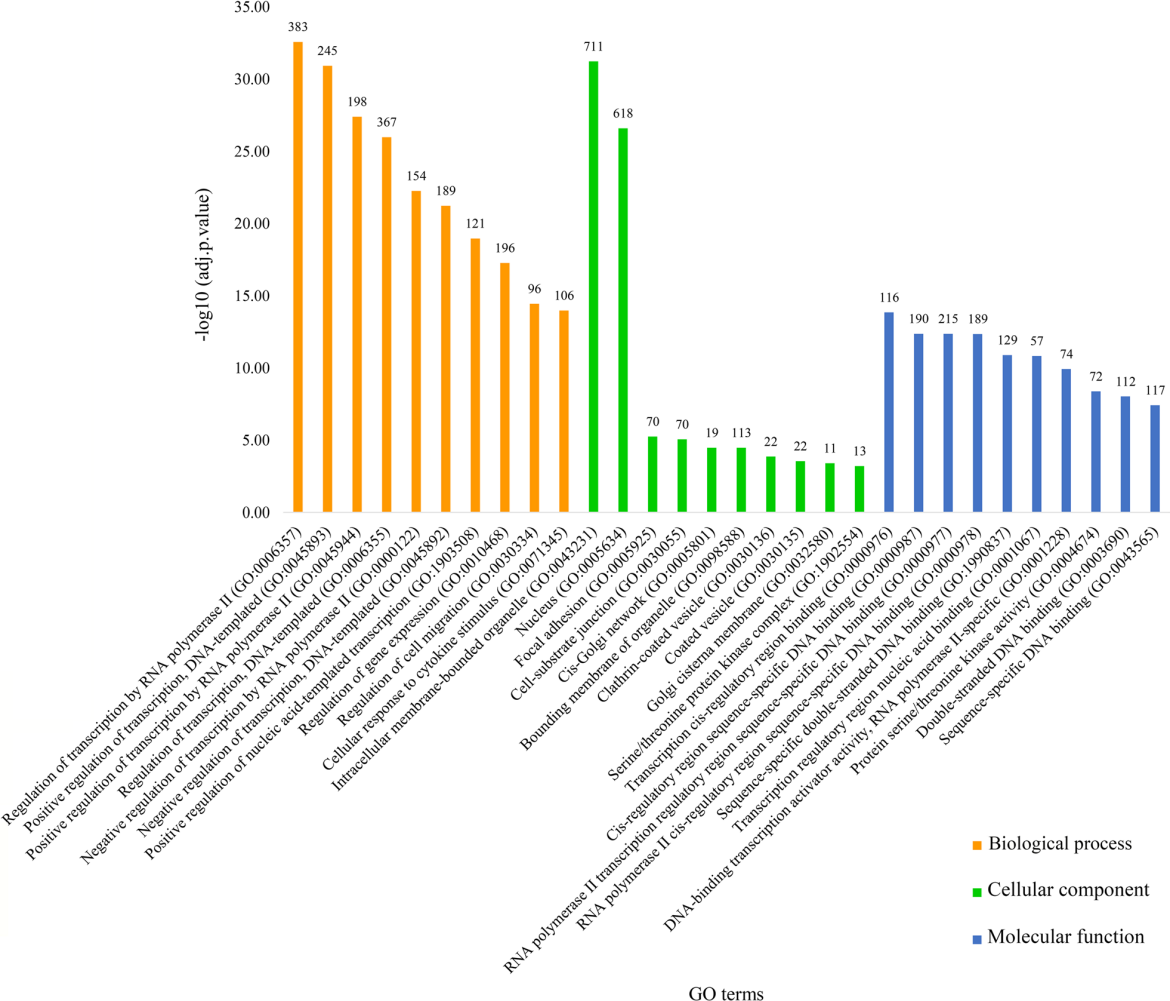
Gene Ontology (GO) analysis of mRNAs (target genes) from the miRNA-mRNA network. Top 10 GO terms in each category and their -log10 (adjusted p-value) are represented. The numbers above each GO term represent the number of mRNAs relating to them

**Fig. 6:**
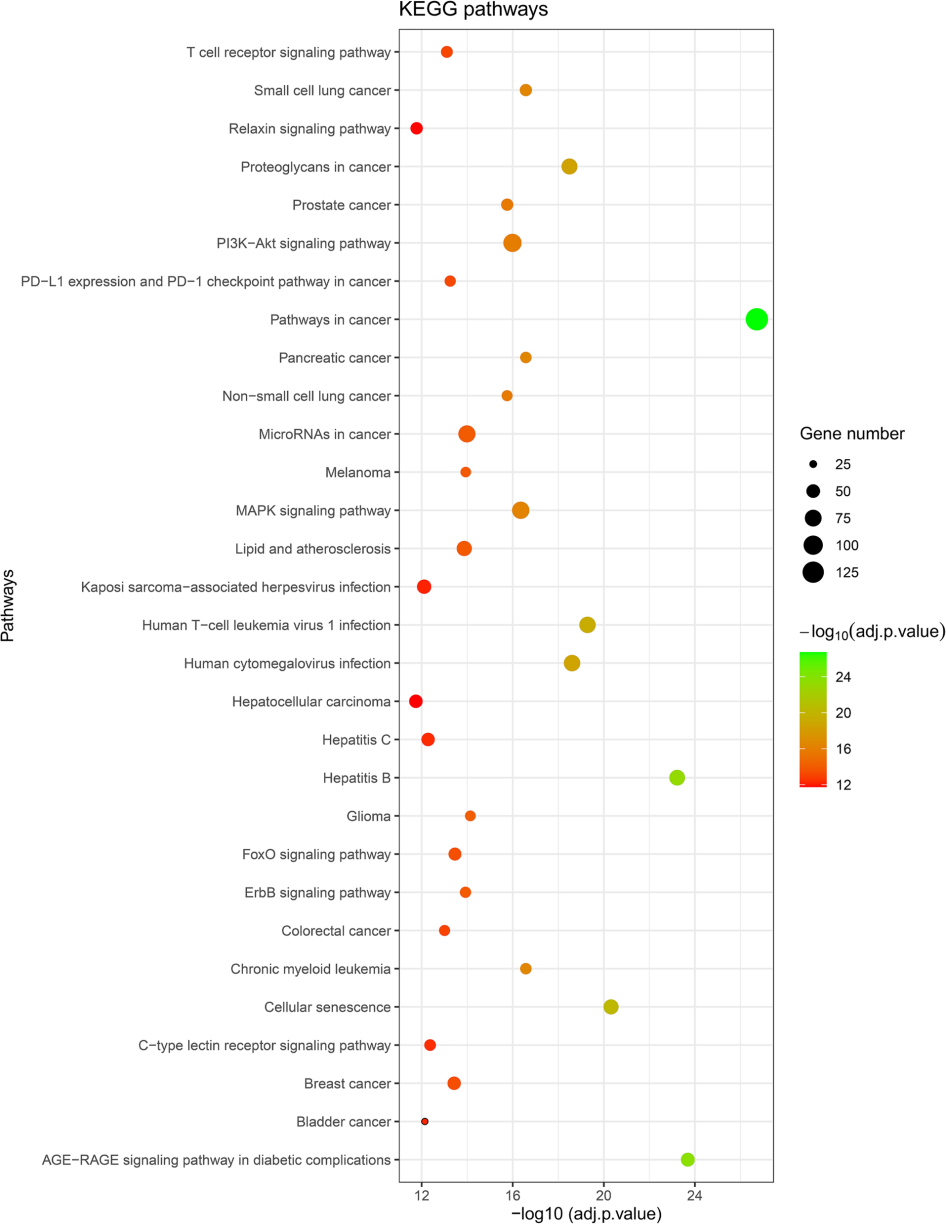
Kyoto Encyclopedia of Genes and Genomes (KEGG) analysis of mRNAs (target genes) from the miRNA-mRNA network. Top 30 most significantly enriched KEGG pathways related to the target genes of miRNAs involved in the odontogenic/osteogenic differentiation of hDP-MSCs are presented. The plot is based on decreasing order of − log10 (adjusted *p* value). The size of circles represents the number of genes involved in each pathway

Out of 46 miRNAs, the multiMiR R package identified target genes (mRNAs) of 31 miRNAs. A miRNA-mRNA network including 1890 nodes (31 miRNAs and 1859 mRNAs) and 2171 edges was then constructed (Fig. 4). The network has been made available on the Network Data Exchange (https://www.ndexbio.org/#/network/3a7985b0-cfb7-11ec-b397-0ac135e8bacf?accesskey=f3527730ff25ef6ad91254fb3443e1e96b0a9e49abee3324410b5f17c1b46af6), a database and online community for sharing and collaborative development of network models [65]. GO and KEGG enrichment analyses revealed important GO terms and KEGG pathways related to the mRNAs from the miRNA-mRNA network. The 30 most significant KEGG pathways and GO terms (10 terms in each GO group including BP, CC and MF) were reported (Figs. 5 and 6). The complete lists of identified significant KEGG pathways and GO terms, and their related genes have been provided in the Mendeley Data repository (https://doi.org/10.17632/ydhrmf2869.1).

Among the KEGG pathways, pathways in cancer, AGE-RAGE signaling pathway in diabetic complications, hepatitis B, cellular senescence and human T-cell leukemia virus 1 infection were the five most significantly enriched. Regulation of transcription by RNA polymerase II (GO:0006357), intracellular membrane-bounded organelle (GO:0043231) and transcription cis-regulatory region binding (GO:0000976) were the most significantly enriched GO terms related to BP, CC and MF, respectively.

**Fig. 7:**
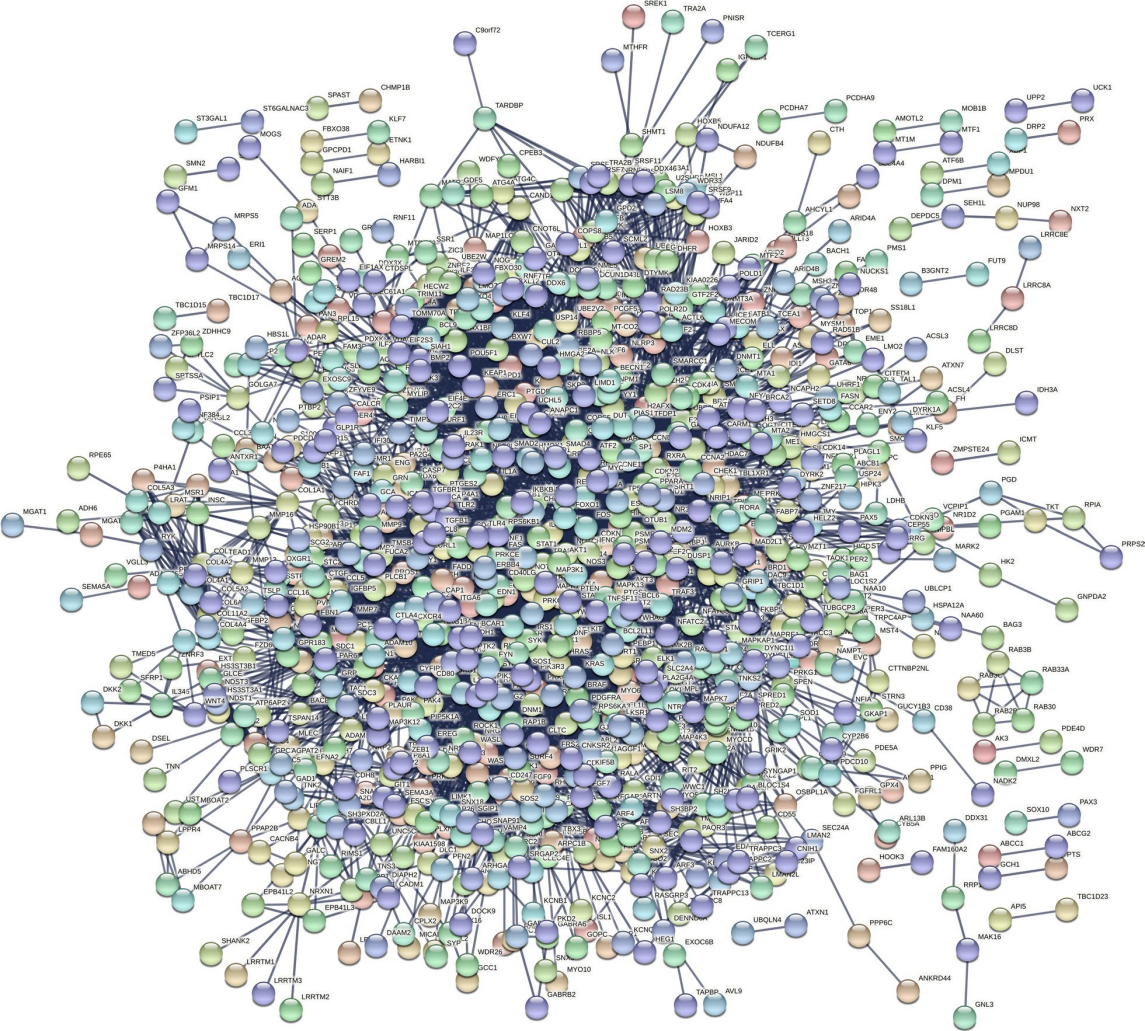
Protein–protein interaction (PPI) network. The interactions among the mRNAs from the miRNA-mRNA network are represented. The thickness of the edges indicates the confidence of each interaction. High-resolution network is available on https://www.ndexbio.org/#/network/f5419fb1-cfc8-11ec-b397-0ac135e8bacf?accesskey=a75d2b627c99900811ff1775a16460d225fc31d281775566da8bc30d95e40332

**Fig. 8:**
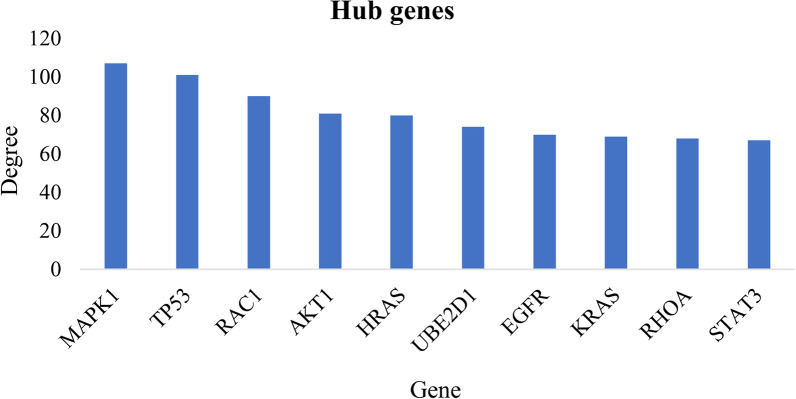
Ten hub genes from the protein–protein interaction (PPI) network based on degree centrality

Furthermore, a PPI network including 1016 nodes and 6199 edges was established based on the mRNAs from the miRNA-mRNA network and ten hub proteins (based on degree centrality) from the network were identified (Figs. 7 and 8). The complete list of interactions among proteins has been provided in the Mendeley Data repository (https://doi.org/10.17632/ydhrmf2869.1). The hub proteins included MAPK1, TP53, RAC1, AKT1, HRAS, UBE2D1, EGFR, KRAS, RHOA and STAT3. Moreover, the network is available on the Network Data Exchange (https://www.ndexbio.org/#/network/f5419fb1-cfc8-11ec-b397-0ac135e8bacf?accesskey=a75d2b627c99900811ff1775a16460d225fc31d281775566da8bc30d95e40332).

## Discussion

Since the discovery of miRNAs and their roles as regulatory molecules, a variety of studies have investigated and highlighted their role in the differentiation of stem cells, which are key components in the field of regenerative therapies [66]. hDP-MSCs, as promising cell sources in this field, have recently attracted increasing attention [4]. In this systematic review, a panel of important miRNAs regulating the differentiation of hDP-MSCs to odontogenic/osteogenic, myogenic, angiogenic and neuronal lineages was collected.

Included studies mainly focused on odontoblastic/osteoblastic differentiation. Odontogenesis and osteogenesis are both classified as mineralized tissues formation with several properties in common. Odontoblasts and osteoblasts express similar differentiation markers such as ALP, RUNX2 and COL1 [67]. Similar signaling pathways such as Wnt/β-catenin are involved in their differentiation [68]. The same growth factors and induction medium are used to differentiate stem cells to odontoblastic and osteoblastic lineages [36, 58]. Furthermore, similar techniques such as alizarin red staining and ALP staining are used to assess differentiation to both [36, 58].

Among the miRNAs identified from the group I studies, hsa-miR-140-5p, hsa-miR-218 and hsa-miR-143 were more frequently reported and are discussed below.

Lu et al. [51] found that overexpression of hsa-miR-140-5p knocks down the expression of odontogenic differentiation markers such as DMP1 and DSPP, and suppresses odontoblastic differentiation through Wnt/β-Catenin signaling pathway. Numerous studies support Wnt/β-Catenin as a key pathway in stem cell proliferation, self-renewal and differentiation [69, 70]. Another study [52] reported that hsa-miR-140-5p promoted proliferation and suppressed odontogenic differentiation of hDPSCs via lipopolysaccharide/Toll-like receptor 4 pathway by targeting TLR-4, a significant regulator of hDPSCs. Other studies investigating odontogenic/osteogenic differentiation revealed other target genes of hsa-miR-140-5p including *FGF9*, *BMP2* and *GIT2* [36, 43, 50]. BMP2 and FGF9 are well recognized as crucial growth factors involving in odontogenic/osteogenic differentiation [17, 60, 71]. Besides the suppressive effect of hsa-miR-140-5p on odontogenic/osteogenic differentiation, Liu et al. [43] reported that it also inhibits direct differentiation of SHEDs to neuronal lineage by down-regulating BMP2 expression. hsa-miR-218 is another frequently reported miRNA having inhibitory effects on odontogenic/osteogenic differentiation. The results of a study by Gay et al. [55] revealed that hsa-miR-218 targets RUNX2, which is a master regulator of mineralized tissue formation such as odontogenesis, leading to decreased mineralization of DPSCs. Chang et al. [54] transfected hsa-miR-218 into the hDPSCs and reported that its inhibitory effect occurs through activation of MAPK, especially through the ERK1/2 pathway. A previous study [72] reported that ERK1/2 signaling converges at RUNX2 to control odontogenic differentiation. Furthermore, another study [53] reported that hsa-miR-218 restrains the proliferation and osteoblastic differentiation of hDPSCs through the repression of lncRNA-CCAT1. hsa-miR-143 family was another frequently reported miRNA inhibiting odontogenic/osteogenic differentiation. Wang et al. [57] suggested that inhibition of hsa-miR-143-5p enhances odontogenic differentiation through activation of p38 MAPK signaling pathway through up-regulation of *MAPK14*. Previous studies have suggested that p38 MAPK signaling pathway plays a role in regulation of the odontogenic differentiation of hDPSCs [14, 73]. Yang et al. [56] identified that hsa-miR-143-3p regulates OPG/RANKL signaling through targeting RANK. Another study [58] reported that hsa-miR-143 binds to 3′-UTR of *TNF-α* and inactivates the NF-κB signaling pathway, consequently impairing hDPSCs differentiation to osteoblast-like cells. Additionally, Li et al. [47], reported that hsa-miR-143 along with hsa-miR-135 attenuates the differentiation of hDPSCs to the skeletal myogenic lineage.

Regarding the group II, the miRNAs reported in at least two studies have been previously identified as important regulators of cell differentiation [74–77], among which the most frequently investigated in previous studies are as follows: hsa-miR-100 was reported as an important endogenous suppressor of bone morphogenic protein-induced osteoblastic differentiation by down-regulating Smad1 [74]. Regarding hsa-miR-146a-5p, Qiu et al. [75] concluded that it enhances DPSCs differentiation to odontogenic/osteogenic lineage by suppressing the Notch pathway. Gao et al. [76] identified that hsa-miR-130b is overexpressed in osteogenically differentiated MSCs from bone marrow. Shao et al. [77] discovered that hsa-miR-122 up-regulated OSX, RUNX2, OCN, COL1 and BMP2 expression resulting in enhanced osteoblastic differentiation of bone marrow-derived mesenchymal stem cells. Zhang et al. [78] identified that hsa-miR-20b is up-regulated during differentiation of stem cells derived from human adipose tissues toward osteogenic lineage. hsa-miR-483-3p promotes osteoblastic differentiation of bone marrow mesenchymal stem cells by binding to the 3′-UTR of STAT1, leading to increased activity of RUNX2 and its nuclear translocation [79, 80]. hsa-miR-34a promotes odontogenic/osteogenic differentiation of stem cells from the apical papilla of the tooth through inhibition of Notch signaling pathway by attenuating NOTCH2 and HES1 expression [81]. In addition to the Notch pathway, hsa-miR-34a has important functions in differentiation of dental papilla cells through TGF-β signaling pathway [82]. hsa-miR-27a-5p has been shown to be overexpressed in exosomes obtained from odontogenically differentiated dental pulp stem cells compared to undifferentiated cells promoting odontogenic differentiation through TGFβ1/smads signaling pathway [16]. Zhang et al. [83] disclosed that inhibition of hsa-miR-135 could improve cell viability and osteoblastic differentiation via activating JAK2/STAT3 signaling pathway. On the contrary, Si et al. [84] reported that hsa-miR-135b-5p activates the HIPPO signaling pathway and promotes osteogenesis by targeting *LATS1* and *MOB1B*, negative regulatory factors of the HIPPO pathway.

As there was high heterogeneity among the studied miRNAs, bioinformatic analyses were conducted to compile these data. Firstly, the constructed miRNA-mRNA network revealed comprehensive interactions in odontogenic/osteogenic differentiation of hDP-MSCs. Furthermore, KEGG pathway analysis revealed the signaling pathways potentially involved in odontogenic/osteogenic differentiation of hDP-MSCs. Among the 30 most significant pathways, there were several important pathways with identified roles in odontogenic/osteogenic differentiation such as MAPK, PI3K-Akt and FoxO.

The MAPK signaling pathway is one of the most frequently discussed in odontogenic differentiation and has been identified to be correlated with cellular differentiation [85]. It has been reported that the MAPK signaling pathway is involved in lipopolysaccharide-mediated odontogenic/osteogenic differentiation of stem cells from the apical papilla [86]. miRNAs, by binding to their target RNAs, can affect the MAPK signaling pathway and odontogenic differentiation. miRNA Let-7c, through targeting insulin-like growth factor 1 receptor (IGF-1R), affects the MAPK signaling pathway and inhibits odontogenic/osteogenic differentiation of hDP-MSCs [87]. Down-regulation of hsa-miR-143-5p which targets MAPK14 increases activation of the p38 MAPK signaling pathway and induces odontoblastic differentiation of hDPSCs [57].

The PI3K/Akt signaling pathway can promote osteoblastic differentiation of human mesenchymal stem cells [88]. In a study conducted by Zhang et al. [89], it was disclosed that the PI3K/AKT signaling pathway can induce odontogenic differentiation of hDPSCs. Xiao et al. [90] found 223 differentially expressed proteins between differentiated and undifferentiated DPSCs. KEGG analysis revealed that the PI3K-Akt signaling pathway significantly correlates with these proteins. Also, a previous study [91] highlighted the existence of a cross talk between PI3K/Akt and Wnt/β-Catenin pathway, a well-known signaling involved in odontogenic/osteogenic differentiation.

Forkhead box O (FoxO) has a crucial role in regulation of cellular differentiation [92]. Essential roles of FoxO in osteogenic differentiation have been identified [93]. Chen et al. [15] utilized microarray and identified differentially expressed lncRNAs, mRNAs and miRNAs in odontogenic differentiated compared to undifferentiated human dental pulp stem cells. They constructed a competing endogenous RNA (ceRNA) network, and KEGG pathway analysis of the differentially expressed mRNAs revealed that the FoxO signaling pathway is the most significant pathway involved in odontogenic differentiation. In another study [94], it was revealed that the FoxO signaling pathway significantly correlates with the target genes of differentially expressed circRNAs in odontogenic differentiation of hDPSCs.

GO enrichment analysis revealed underlying biological terms of the odontogenic/osteogenic differentiation of hDP-MSCs. The most significantly enriched GO terms were regulation of transcription by RNA polymerase II (GO:0006357), intracellular membrane-bounded organelle (GO:0043231) and transcription cis-regulatory region binding (GO:0000976) within the categories of BP, CC and MF, respectively. Regulation of transcription by RNA polymerase II is related to *MAPK7*, *MAPK14*, *BMP2*, *BMP3*, *SMAD2*, *SMAD4*, *SMAD5*, *STAT1*, *STAT3*, *STAT5A*, *STAT5B*, *FOXO1* and *FOXO3* genes which are all previously reported to be involved in odontogenic/osteogenic differentiation [15, 16, 57, 60, 77, 80, 81]. Likewise, intracellular membrane-bounded organelle and transcription cis-regulatory region binding are also associated with odontogenic/osteogenic-related genes such as *SMAD2*, *SMAD4*, *STAT1* and *STAT3* [16, 59, 80].

Finally, ten hub proteins of the PPI network were identified most of which are well-known proteins involved in regulating cell cycle, proliferation, migration and differentiation [85, 89, 95].

## Strengths, limitations and future perspectives

To the best of our knowledge, the current study is the first that systematically reviews the miRNAs with identified roles in the differentiation of hDP-MSCs. Of note, the major strength of the current systematic review is that bioinformatic analyses were conducted which add a new layer of information to the previously studied miRNAs in the differentiation of hDP-MSCs as it identified signaling pathways and other cellular and molecular characteristics influenced by the union of miRNAs during the differentiation process. These findings provide a deeper view in the field of studying the significance of miRNAs in the differentiation of hDP-MSCs. Another strength of the current systematic review is that we included the studies which investigated expression profile changes of miRNAs during the differentiation process of hDP-MSCs by utilizing high-throughput techniques along with those experimentally validated the effect of specific miRNA(s) on this process. In the other words, high-throughput-based studies are hypothesis-free taking a non-biased approach and are more likely to come up with the introduction of novel miRNAs involved in the differentiation of hDP-MSCs.

The limitations of the study were, firstly, publication bias which may exist as unpublished studies with negative outcomes might have been missed. Thus, overestimation of the effect of miRNAs on the differentiation of hDP-MSCs may have occurred. Secondly, the focus was on miRNAs and their interactions with mRNAs, however, miRNAs interact with other types of RNAs such as lncRNAs and circRNAs. Therefore, future studies should be conducted to determine the role of other types of RNAs along with miRNAs in the differentiation of hDP-MSCs. The third limitation was that among the included studies, those applying low-throughput and hypothesis-driven strategies (those experimentally validated the effect of a specific miRNA) outnumbered the high-throughput-based studies.

Understanding the contribution of miRNAs to the differentiation of hDP-MSCs is still in its infancy. Although applying high-throughput techniques including next generation sequencing and microarray have introduced several putative miRNAs which are involved in the differentiation of hDP-MSCs, most of them are still awaiting further confirmation and functional analysis. Additionally, the role of miRNAs in various biological processes and diseases has drawn attention to the potential application of these molecules as gene therapies. Therefore, future studies are warranted to investigate the possibility of their clinical utilization in the field of regenerative medicine.

## Conclusions

Understanding the regulatory mechanisms underlying the differentiation of hDP-MSCs is integral for their therapeutic application. The current review implies that specific miRNAs and signaling pathways are involved in the regulation of hDP-MSCs differentiation. hsa-miR-140-5p, hsa-miR-143 family and hsa-miR-218 were the most frequently reported miRNAs suppressing odontogenic/osteogenic differentiation. MAPK, PI3K-Akt and FoxO were identified as key signaling pathways involved in the differentiation of hDP-MSCs. These findings support the potential application of miRNAs to regulate the directed differentiation of hDP-MSCs in the field of stem cell-based regenerative therapies.

## Supplementary Information

**Additional file 1: Table S1.** Search strategy and number of records retrieved from each database.

**Additional file 2: Table S2.** Excluded studies and reason of exclusion.

**Additional file 3: Table S3.** Complete list of differentially expressed miRNAs during odontogenic/osteogenic differentiation of hDP-MSCs retrieved from the results of group II studies.

## Data Availability

The datasets generated during the current study are openly available in the Mendeley Data repository at https://doi.org/10.17632/ydhrmf2869.1 [96].

## Abbreviations

3′-UTR: 3′ Untranslated region
ALP: Alkaline phosphatase
BMP2: Bone morphogenetic protein-2
BP: Biological process
CC: Cellular component
COL1: Collagen-1
DMP1: Dentine matrix protein 1
DSPP: Dentine sialophosphoprotein
ERk: Extracellular receptor kinase
FGF9: Fibroblast growth factor-9
FoxO: Forkhead box transcription factors
GO: Gene Ontology
hDP-MSC: Human dental pulp-derived mesenchymal stem cell
hDPSC: Human dental pulp stem cells
KEGG: Kyoto Encyclopedia of Genes and Genomes
MAPK: Mitogen-activated protein kinase
MF: Molecular functions
miRNA: MicroRNA
mRNAs: Messenger RNA
MSC: Mesenchymal stem cell
NF-kB: Nuclear factor kappa B
OCN: Osteocalcin
OPG: Osteoprotegerin
OPG: Osteoprotegerin
OPN: Osteopontin
OSX: Osterix
PI3K-Akt: Phosphoinositide 3-kinase-Akt
PPI: Protein–protein interaction
PRISMA: Preferred Reporting Items for Systematic Reviews and Meta-Analyses
qRT-PCR: Quantitative reverse transcription polymerase chain reaction
RANKL: Receptor activator of NF-kB ligand
RUNX2: Runt-related transcription factor-2
SHED: Stem cell from human exfoliated deciduous teeth
TGF: Transforming growth factor
TLR-4: Toll-like receptor-4
