# Supplementary material for: MicroRNAs-mediated regulation of the differentiation of dental pulp-derived mesenchymal stem cells: a systematic review and bioinformatic analysis
Source: Stem Cell Res Ther. 2023 Apr 11;14:76. doi: 10.1186/s13287-023-03289-5 (PMC10088330; doi:10.1186/s13287-023-03289-5)
Supplement: Supplementary file 1 — Additional file 1: Table S1. Search strategy and number of records retrieved from each database. [file 13287_2023_3289_MOESM1_ESM.docx]

| Table S1  Search strategy and number of records retrieved from each database | | |  |
| --- | --- | --- | --- |
| Database | **Search query** | **Number of retried records** | |
| PubMed | ("micro RNA"[Title/Abstract] OR "micro RNAs"[Title/Abstract] OR "micro-RNA"[Title/Abstract] OR "micro-RNAs"[Title/Abstract] OR microRNA OR microRNAs OR miRNA OR miRNAs OR "micro ribonucleic acid"[Title/Abstract] OR "micro ribonucleic acids"[Title/Abstract] OR miR) AND ((("dental pulp"[Title/Abstract] OR pulp OR "exfoliated deciduous"[Title/Abstract]) AND ("stem cell"[Title/Abstract] OR "stromal cell"[Title/Abstract] OR "progenitor cell"[Title/Abstract] OR "stem cells"[Title/Abstract] OR "stromal cells"[Title/Abstract] OR "progenitor cells"[Title/Abstract])) OR DPSCs OR hDPSCs OR DPSC OR hDPSC) | 98 | |
| Web of Science | TS= (micro-RNA OR microRNA OR miRNA OR micro-ribonucleic-acid OR miR) AND TS= (((dental-pulp OR pulp OR (exfoliated NEAR/3 deciduous)) NEAR/5 (stem-cell OR stromal-cell OR progenitor-cell)) OR DPSC OR hDPSC) | 98 | |
| Scopus | TITLE-ABS-KEY (("micro RNA" OR "micro-RNA" OR microRNA OR miRNA OR "micro ribonucleic acid" OR miR) AND ((("dental pulp" OR pulp OR "exfoliated deciduous") W/5 ("stem cell" OR "stromal cell" OR "progenitor cell")) OR DPSC OR hDPSC)) | 111 | |
| Embase | (micro-RNA OR microRNA OR miRNA OR micro-ribonucleic-acid OR miR) AND (((dental-pulp OR pulp OR exfoliated-deciduous) NEAR/5 (stem-cell OR stromal-cell OR progenitor-cell OR stem-cells OR stromal-cells OR progenitor-cells)) OR DPSC OR hDPSC) | 113 | |
| Cochrane Library | (("micro RNA" OR "micro-RNA" OR microRNA OR miRNA OR "micro ribonucleic acid" OR miR)):ti,ab,kw AND ((("dental pulp" OR pulp OR "exfoliated deciduous") NEAR/5 ("stem cell" OR "stromal cell" OR "progenitor cell" )) OR DPSC OR hDPSC):ti,ab,kw | 0 | |
| ProQuest | (micro-RNA OR microRNA OR miRNA OR micro-ribonucleic-acid OR miR) AND (((dental-pulp OR pulp OR (exfoliated N/3 deciduous)) N/5 (stem-cell OR stromal-cell OR progenitor-cell)) OR DPSC OR hDPSC) | 234 | |
| OpenGrey | (micro RNA* OR micro-RNA* OR microRNA* OR miRNA* OR micro ribonucleic acid* OR miR) AND (((dental pulp OR pulp OR exfoliated deciduous) AND (stem cell* OR stromal cell* OR progenitor cell*)) OR DPSC* OR hDPSC*) | 0 | |
| WorldCat | su:(micro RNA* OR micro-RNA* OR microRNA* OR miRNA* OR micro ribonucleic acid* OR miR) AND (((dental pulp OR pulp OR exfoliated deciduous) AND (stem cell* OR stromal cell* OR progenitor cell*)) OR DPSC* OR hDPSC*) | 12 | |
| Google Scholar | microRNAs AND differentiation AND dental pulp AND stem cells | First 100 hits | |
