# Supplementary material for: MicroRNAs-mediated regulation of the differentiation of dental pulp-derived mesenchymal stem cells: a systematic review and bioinformatic analysis
Source: Stem Cell Res Ther. 2023 Apr 11;14:76. doi: 10.1186/s13287-023-03289-5 (PMC10088330; doi:10.1186/s13287-023-03289-5)
Supplement: Supplementary file 2 — Additional file 2: Table S2. Excluded studies and reason of exclusion. [file 13287_2023_3289_MOESM2_ESM.docx]

| Table S2  Excluded studies and reason of exclusion | |
| --- | --- |
| Reason of exclusion | **Studies** |
| Studies which applied dental pulp-derived stem cells from other species rather than human | (1-10) |
| Not an original study | (11-17) |
| Studies which were not in English | (18, 19) |
| Full text not accessible | (20) |
| Retracted study | (21, 22) |

**References**

1. Yao S, Li C, Budenski AM, Li P, Ramos A, Guo S. Expression of microRNAs targeting heat shock protein B8 during in vitro expansion of dental pulp stem cells in regulating osteogenic differentiation. *Arch Oral Biol*. 2019;107:104485.

2. Nozaki T, Ohura K. Inhibition of miR-183 Induces Insulin in Dental Pulp Cells. *J Hard Tissue Biol*. 2017;26:319-22.

3. Park M-G, Kim J-S, Park S-Y, Lee SA, Kim H-J, Kim CS, et al. MicroRNA-27 promotes the differentiation of odontoblastic cell by targeting APC and activating Wnt/β-catenin signaling. *Gene*. 2014;538(2):266-72.

4. Xie Y, Shen G. MicroRNA-139-5p elevates skeletal myogenic differentiation of human adult dental pulp stem cells through Wnt/β-catenin signaling pathway. *Exp Ther Med*. 2018;16(4):2835-42.

5. Wang YL, Zuo CR, Wang J, Yang K, Wang SR, Zhang XP. MicroRNA-210 promotes the proliferation and odontogenic differentiation of rat dental pulp stem cells. *Chinese Journal of Tissue Engineering Research*. 2019;23(25):4004-10.

6. Sun Q, Liu H, Lin H, Yuan G, Zhang L, Chen Z. MicroRNA-338-3p promotes differentiation of mDPC6T into odontoblast-like cells by targeting Runx2. *Mol Cell Biochem*. 2013;377(1-2):143-9.

7. Heair HM, Kemper AG, Roy B, Lopes HB, Rashid H, Clarke JC, et al. MicroRNA-665 Regulates Dentinogenesis through MicroRNA-Mediated Silencing and Epigenetic Mechanisms. *Mol Cell Biol*. 2015;35:3116–30.

8. Yuan H, Zhao H, Wang J, Zhang H, Hong L, Li H, et al. MicroRNA let-7c-5p promotes osteogenic differentiation of dental pulp stem cells by inhibiting lipopolysaccharide-induced inflammation via HMGA2/PI3K/Akt signal blockade. *Clin Exp Pharmacol Physiol*. 2019;46(4):389-97.

9. Liu H, Lin H, Zhang L, Sun Q, Yuan G, Zhang L, et al. miR-145 and miR-143 Regulate Odontoblast Differentiation through TargetingKlf4andOsxGenes in a Feedback Loop. *J Biol Chem*. 2013;288(13):9261-71.

10. Nozaki T, Ohura K. Regulation of miRNA during direct reprogramming of dental pulp cells to insulin-producing cells. *Biochem Biophys Res Commun*. 2014;444(2):195-8.

11. Bayarsaihan D. Focus: Epigenetics: Deciphering the Epigenetic Code in Embryonic and Dental Pulp Stem Cells. *The Yale Journal of Biology and Medicine*. 2016;89(4):539.

12. Gene expression profile during odontogenic differentiation was analyzed using a microarray (miRnA) [Internet]. 2019 [cited 2020-07-23]. Available from: <Go to ISI>://DRCI:DATA2020158019732870

<http://www.ncbi.nlm.nih.gov/geo/query/acc.cgi?acc=GSE138180>.

13. Passos G. Identification of mirna during osteoblast differentiation of human dental pulp stem cells. *ArrayExpress Archive*. 2014.

14. Passos G. Identification of networks mirna-mrna interactions during osteoblast differentiation of human dental pulp stem cells. *ArrayExpress Archive*. 2014.

15. Ren C, Liu Y, Xu N, Shao M, He J, Li X. Non-coding rnas in human dental pulp stem cells: Regulations and mechanisms. *Chinese Journal of Tissue Engineering Research*. 2020;24(7):1130-7.

16. Fang F, Zhang K, Chen Z, Wu B. Noncoding RNAs: new insights into the odontogenic differentiation of dental tissue-derived mesenchymal stem cells. *Stem Cell Res Ther*. 2019;10(1):297.

17. Sehic A, Tulek A, Khuu C, Nirvani M, Sand LP, Utheim TP. Regulatory roles of microRNAs in human dental tissues. *Gene*. 2017;596:9-18.

18. Xuan D, Fang Y, Xiqing T, Yan Z. Microrna-20 regulates directional differentiation of stem cells from human exfoliated deciduous teeth. *Chinese Journal of Tissue Engineering Research*. 2019;23(21):3329-35.

19. Wenhui W, Lei G, Cuixia J, Yuling Y, Dongdong Y, Nan W. MicroRNA-431 effects on the differentiation and proliferation of human dental pulp stem cells. *Chinese Journal of Tissue Engineering Research*. 2019;23(21):3323-8.

20. Miao J, Zhang D, Zhang S, Zhao Q, Liu C, Li L, et al. MicroRNA-320c regulates the proliferation and differentiation of human dental pulp stem cells. *Journal of Biomaterials and Tissue Engineering*. 2018;8(4):530-6.

21. Yu D, Zhao X, Cheng J-Z, Wang D, Zhang H-H, Han G-H. Downregulated microRNA-488 enhances odontoblast differentiation of human dental pulp stem cells via activation of the p38 MAPK signaling pathway. *J Cell Physiol*. 2019;234(2):1442-51.

22. Zhan F-L, Liu X-Y, Wang X-B. The Role of MicroRNA-143-5p in the Differentiation of Dental Pulp Stem Cells into Odontoblasts by Targeting Runx2 via the OPG/RANKL Signaling Pathway. *J Cell Biochem*. 2018;119(1):536-46.
