# Supplementary material for: MicroRNAs-mediated regulation of the differentiation of dental pulp-derived mesenchymal stem cells: a systematic review and bioinformatic analysis
Source: Stem Cell Res Ther. 2023 Apr 11;14:76. doi: 10.1186/s13287-023-03289-5 (PMC10088330; doi:10.1186/s13287-023-03289-5)
Supplement: Supplementary file 3 — Additional file 3: Table S3. Complete list of differentially expressed miRNAs during odontogenic/osteogenic differentiation of hDP-MSCs retrieved from the results of group II studies. [file 13287_2023_3289_MOESM3_ESM.docx]

| Table S3  Complete list of differentially expressed miRNAs during odontogenic/osteogenic differentiation of hDP-MSCs from the results of group II studies. | | |
| --- | --- | --- |
| Study | **Up-regulated miRNAs** | **Down-regulated miRNAs** |
| Liu et al, 2020 (1) | hsa-miR-138-5p, hsa-miR-20a-3p, hsa-miR-324-5p, hsa-miR-1185-5p, hsa-miR-500a-5p, hsa-miR-548j-3p, hsa-miR-4524a-3p, hsa-miR-4524b-5p, hsa-miR-660-3p, hsa-miR-33a-3p, hsa-miR-378a-5p, hsa-miR-377-3p, hsa-miR-381-5p, hsa-miR-592, hsa-miR-1197, hsa-miR-483-3p, hsa-miR-192-5p, hsa-miR-18a-5p, hsa-miR-582-5p, hsa-miR-16-1-3p, hsa-miR-301b-3p, hsa-miR-32-5p, hsa-miR-1277-5p, hsa-let-7i-3p, hsa-miR-1307-5p, hsa-miR-194-5p, hsa-miR-660-5p, hsa-miR-193a-3p, hsa-miR-100-3p, hsa-miR-500b-5p, hsa-miR-3611, hsa-miR-15a-3p, hsa-miR-137, hsa-miR-514a-3p, hsa-miR-3065-5p, hsa-miR-338-3p, hsa-miR-532-3p, hsa-miR-2355-5p, hsa-miR-21-3p, hsa-miR-212-3p, hsa-miR-95-3p, hsa-miR-4773, hsa-miR-188-5p, hsa-miR-1247-3p, hsa-miR-579-3p, hsa-let-7f-2-3p, hsa-miR-190b, hsa-miR-490-3p, hsa-miR-27a-5p, hsa-miR-509-5p, hsa-miR-194-3p, hsa-miR-362-3p, hsa-miR-33a-5p, hsa-miR-19b-3p, hsa-miR-19a-3p, hsa-miR-1247-5p, hsa-miR-597-5p, hsa-miR-1260b, hsa-miR-5690, hsa-miR-146a-3p, hsa-miR-4454, hsa-miR-146a-5p | hsa-miR-335-3p, hsa-miR-218-5p, hsa-miR-383-5p, hsa-miR-335-5p, hsa-miR-200a-5p, hsa-miR-27b-5p, hsa-miR-877-5p, hsa-miR-146b-3p, hsa-miR-584-5p, hsa-miR-342-5p, hsa-miR-4461, hsa-miR-370-3p, hsa-miR-3529-3p, hsa-miR-7-5p, hsa-miR-23b-5p, hsa-miR-205-5p, hsa-miR-210-5p, hsa-miR-7974, hsa-miR-296-3p, hsa-miR-877-3p, hsa-miR-452-5p, hsa-miR-1303, hsa-miR-99a-5p, hsa-let-7c-3p, hsa-miR-873-3p, hsa-miR-224-3p, hsa-miR-4521, hsa-miR-3180, hsa-miR-3180-3p, hsa-miR-4473, hsa-miR-433-3p, hsa-miR-605-3p, hsa-miR-328-3p, hsa-miR-6720-3p, hsa-miR-671-5p, hsa-miR-185-3p, hsa-miR-4423-3p, hsa-miR-1226-3p, hsa-miR-187-3p, hsa-miR-543, hsa-miR-766-3p, hsa-miR-4485-3p, hsa-miR-6723-5p, hsa-miR-27b-3p, hsa-miR-375, hsa-miR-5002-5p, hsa-miR-1299, hsa-miR-330-3p |
| Chen et al, 2020 (2) | hsa-miR-1266-3p, hsa-miR-1273g-3p, hsa-miR-146a-5p, hsa-miR-188-5p, hsa-miR-2681-5p, hsa-miR-3064-5p, hsa-miR-3074-3p, hsa-miR-3610, hsa-miR-3621, hsa-miR-4253, hsa-miR-4522, hsa-miR-4767, hsa-miR-491-3p, hsa-miR-5196-5p, hsa-miR-5684, hsa-miR-605-5p, hsa-miR-662, hsa-miR-671-5p, hsa-miR-6746-5p, hsa-miR-6779-5p, hsa-miR-6806-5p, hsa-miR-6817-3p, hsa-miR-7114-3p, hsa-miR-9500 | hsa-let-7f-2-3p, hsa-miR-101-3p, hsa-miR-1203, hsa-miR-1238-5p, hsa-miR-128-2-5p, hsa-miR-1288-5p, hsa-miR-129-5p, hsa-miR-132-3p, hsa-miR-132-5p, hsa-miR-136-3p, hsa-miR-138-1-3p, hsa-miR-140-3p, hsa-miR-141-3p, hsa-miR-143-3p, hsa-miR-145-3p, hsa-miR-145-5p, hsa-miR-1468-3p, hsa-miR-146b-5p, hsa-miR-153-3p, hsa-miR-15a-5p, hsa-miR-17-3p, hsa-miR-186-5p, hsa-miR-18a-5p, hsa-miR-18b-5p, hsa-miR-193b-3p, hsa-miR-1972, hsa-miR-19a-3p, hsa-miR-212-5p, hsa-miR-224-5p, hsa-miR-2355-5p, hsa-miR-26a-5p, hsa-miR-26b-5p, hsa-miR-3074-5p, hsa-miR-30b-5p, hsa-miR-32-5p, hsa-miR-337-3p, hsa-miR-339-5p, hsa-miR-33a-3p, hsa-miR-340-5p, hsa-miR-342-3p, hsa-miR-342-5p, hsa-miR-34b-3p, hsa-miR-34c-5p, hsa-miR-3607-3p, hsa-miR-3653-3p, hsa-miR-3658, hsa-miR-369-3p, hsa-miR-374a-5p, hsa-miR-374b-5p, hsa-miR-374c-5p, hsa-miR-376a-3p, hsa-miR-376b-3p, hsa-miR-382-3p, hsa-miR-411-5p, hsa-miR-423-5p, hsa-miR-4421, hsa-miR-4435, hsa-miR-4436a, hsa-miR-4445-3p, hsa-miR-450a-1-3p, hsa-miR-450a-5p, hsa-miR-450b-3p, hsa-miR-455-3p, hsa-miR-4639-5p, hsa-miR-4667-3p, hsa-miR-4668-5p, hsa-miR-4717-3p, hsa-miR-4723-5p, hsa-miR-4730, hsa-miR-491-5p, hsa-miR-5003-5p, hsa-miR-508-3p, hsa-miR-525-5p, hsa-miR-542-3p, hsa-miR-548am-5p, hsa-miR-548d-5p, hsa-miR-5585-3p, hsa-miR-574-3p, hsa-miR-588, hsa-miR-6077, hsa-miR-625-3p, hsa-miR-6512-3p, hsa-miR-6516-3p, hsa-miR-656-3p, hsa-miR-660-3p, hsa-miR-7153-5p, hsa-miR-7160-5p, hsa-miR-744-3p, hsa-miR-889-3p, hsa-miR-99a-3p |
| Hu et al, 2019 (3) | hsa-miR-5100, hsa-miR-27a-5p, hsa-miR-652-3p, hsa-miR-1260a, hsa-miR-1260b, hsa-miR-7f-1-3p, hsa-miR-370-3p | hsa-miR-193a-5p, hsa-miR-4792, hsa-miR-505-3p, hsa-miR-629-5p, hsa-miR-140-3p, hsa-miR-185-5p, hsa-miR-146b-5p, hsa-miR-339-5p, hsa-miR-1246, hsa-miR-107, hsa-miR-320d, hsa-miR-451a, hsa-miR-215-5p, hsa-miR-126-3p, hsa-miR-3687, hsa-miR-31-5p, hsa-miR-210-3p, hsa-miR-1-3p, hsa-miR-10a-5p, hsa-miR-10b-5p, hsa-miR-619-5p |
| Song et al, 2017 (4) | hsa-miR-34a, hsa-miR-20b, hsa-let-7f, hsa-miR-328, hsa-miR-130b, hsa-miR-30e, hsa-miR-503, hsa-miR-214, hsa-miR-937, hsa-miR-138, hsa-miR-122, hsa-miR-200c, hsa-miR-20a, hsa-miR-335, hsa-miR-27a, hsa-miR-338, hsa-miR-126, hsa-miR-19b, hsa-miR-675, hsa-miR-29c, hsa-miR-194-1, hsa-miR-100 | hsa-miR-205, hsa-miR-135b, hsa-miR-142-3p, hsa-miR-192, hsa-miR-15b, hsa-miR-126, hsa-miR-377, hsa-miR-144, hsa-miR-542, hsa-miR-21, hsa-miR-186, hsa-miR-129-3p, hsa-miR-486-3p, hsa-miR-542 |
| Dernowsek et al, 2017 (5) | hsa-miR-145-3p, hsa-miR-30c-5p, hsa-miR-758-3p, hsa-miR-181a-3p, hsa-miR-199-5p, hsa-miR-24-3p, hsa-miR-27a-3p, hsa-miR-27b-3p, hsa-miR-28-5p, hsa-miR-221-3p, hsa-miR-23a-3p | hsa-miR-16-2-3p, hsa-miR-7-5p, hsa-miR-548-3p, hsa-miR-29b-3p, hsa-miR-1914-3p, hsa-miR-29a-3p, hsa-miR-15b-5p, hsa-miR-21-3p, hsa-miR-29b-5p, hsa-miR-1288 |
| Gay et al, 2014 (6) | hsa-mir-708, has-mir-1247 | hsa-miR-502-p, hsa-miR-218, hsa-miR-99a, hsa-miR-210 |
| Hara et al, 2013 (7) | hsa-miR-1260b, hsa-miR-491-3p, hsa-miR-720, hsa-miR-1260, hsa-miR-1280, hsa-miR-4286, hsa-miR-1264, hsa-miR-487b, hsa-miR-3182, hsa-miR-138-1, hsa-miR-205, hsa-miR-1290, hsa-miR-519e, hsa-miR-2115, hsa-miR-767-5p, hsa-miR-4301, hsa-miR-4279, hsa-miR-4275, hsa-miR-4268, hsa-miR-27b, hsa-miR-335, hsa-miR-483-3p, hsa-miR-300, hsa-miR-3646, hsa-miR-1246, hsa-miR-4285, hsa-miR-513a-5p, hsa-miR-1275, hsa-miR-29b, hsa-miR-3175, hsa-miR-3654, hsa-miR-4324, let-7a, hsa-miR-299-5p, hsa-miR-4288, hsa-miR-3935, hsa-miR-3686, hsa-miR-340, hsa-miR-1908, hsa-miR-25, hsa-miR-1469, hsa-miR-4306, hsa-miR-4299, hsa-miR-23a, hsa-miR-149, hsa-miR-29a, let-7b, hsa-miR-4290, hsa-miR-222, let-7c, hsa-miR-3915, hsa-miR-3676, hsa-miR-302a, hsa-miR-221, hsa-miR-3667-5p, hsa-miR-125b, hsa-miR-103a, hsa-miR-3685, hsa-miR-100, hsa-miR-1255a | hsa-miR-607, hsa-miR-200b, hsa-miR-3157-5p, hsa-miR-548g, hsa-miR-1245, hsa-miR-4266, hsa-miR-182, hsa-miR-23b, hsa-miR-1537, hsa-miR-153, hsa-miR-515-5p, hsa-miR-335, hsa-miR-4269, hsa-miR-891b, hsa-miR-3197 |
| Gong et al, 2012 (8) | hsa-miR-20b, hsa-miR-34a, hsa-miR-937, hsa-miR-130b, hsa-miR-100, hsa-miR-335, hsa-miR-944, hsa-miR-17, hsa-miR-562, hsa-miR-338-5p, hsa-miR-122, hsa-miR-521 | hsa-miR-542-5p, hsa-miR-1224-5p, hsa-miR-382, hsa-miR-431, hsa-miR-203, hsa-miR-1225-5p, hsa-miR-486-3p, hsa-miR-517c, hsa-miR-135b, hsa-miR-371-3p |

**References**

1. Liu Z, Xu S, Dao J, Gan Z, Zeng X. Differential expression of lncRNA/miRNA/mRNA and their related functional networks during the osteogenic/odontogenic differentiation of dental pulp stem cells. *J Cell Physiol*. 2020;235(4):3350-61.

2. Chen Z, Zhang K, Qiu W, Luo Y, Pan Y, Li J, et al. Genome-wide identification of long noncoding RNAs and their competing endogenous RNA networks involved in the odontogenic differentiation of human dental pulp stem cells. *Stem Cell Res Ther*. 2020;11(1):114.

3. Hu X, Zhong Y, Kong Y, Chen Y, Feng J, Zheng J. Lineage-specific exosomes promote the odontogenic differentiation of human dental pulp stem cells (DPSCs) through TGFβ1/smads signaling pathway via transfer of microRNAs. *Stem Cell Res Ther*. 2019;10(1):170.

4. Song Z, Chen LL, Wang RF, Qin W, Huang SH, Guo J, et al. MicroRNA-135b inhibits odontoblast-like differentiation of human dental pulp cells by regulating Smad5 and Smad4. *Int Endod J*. 2017;50(7):685-93.

5. Dernowsek JA, Pereira MC, Fornari TA, Macedo C, Assis AF, Donate PB, et al. Posttranscriptional Interaction Between miR-450a-5p and miR-28-5p and STAT1 mRNA Triggers Osteoblastic Differentiation of Human Mesenchymal Stem Cells. *J Cell Biochem*. 2017;118(11):4045-62.

6. Gay I, Cavender A, Peto D, Sun Z, Speer A, Cao H, et al. Differentiation of human dental stem cells reveals a role for microRNA-218. *J Periodontal Res*. 2014;49(1):110-20.

7. Hara ES, Ono M, Eguchi T, Kubota S, Pham HT, Sonoyama W, et al. miRNA-720 controls stem cell phenotype, proliferation and differentiation of human dental pulp cells. *PLoS One*. 2013;8(12):e83545.

8. Gong Q, Wang R, Jiang H, Lin Z, Ling J. Alteration of microRNA expression of human dental pulp cells during odontogenic differentiation. *J Endod*. 2012;38(10):1348-54.
